# Investigating In Vivo Tumor Biomolecular Changes Following Radiation Therapy Using Raman Spectroscopy

**DOI:** 10.1021/acsomega.4c06096

**Published:** 2024-10-09

**Authors:** Varsha Karunakaran, Sina Dadgar, Santosh K. Paidi, April F. Mordi, Whitney A. Lowe, Umme Marium Mim, Jesse D. Ivers, Joel I. Rodriguez Troncoso, Jared A. McPeake, Alric Fernandes, Sanidhya D. Tripathi, Ishan Barman, Narasimhan Rajaram

**Affiliations:** † Department of Biomedical Engineering, University of Arkansas, Fayetteville, Arkansas 72701, United States; ‡ Department of Mechanical Engineering, Johns Hopkins University, Baltimore, Maryland 21218, United States

## Abstract

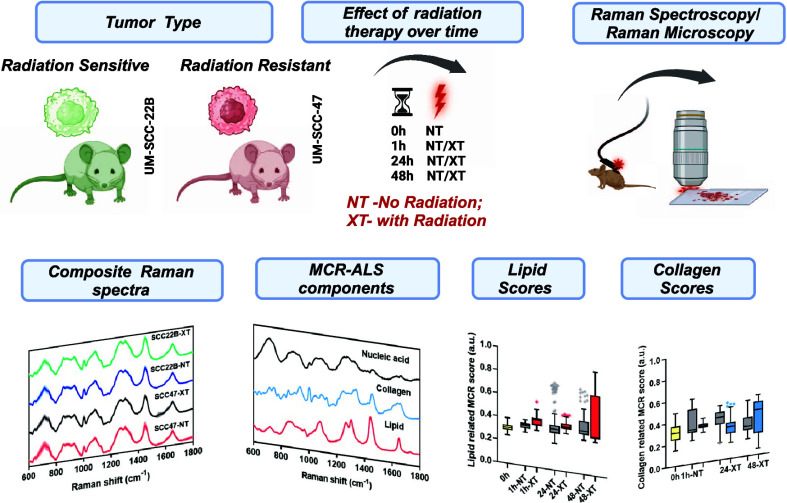

Treatment resistance is a major bottleneck in the success of cancer therapy. Early identification of the treatment response or lack thereof in patients can enable an earlier switch to alternative treatment strategies that can enhance response rates. Here, Raman spectroscopy was applied to monitor early tumor biomolecular changes in sensitive (UM-SCC-22B) and resistant (UM-SCC-47) head and neck tumor xenografts for the first time in in vivo murine tumor models in response to radiation therapy. We used a validated multivariate curve resolution-alternating least-squares (MCR-ALS) model to resolve complex multicomponent Raman spectra into individual pure spectra and their respective contributions. We observed a significant radiation-induced increase in the contributions of lipid-like species (*p* = 0.0291) in the radiation-sensitive UM-SCC-22B tumors at 48 h following radiation compared to the nonradiated baseline (prior to commencing treatment). We also observed an increase in the contribution of collagen-like species in the radiation-resistant UM-SCC-47 tumors at 24 h following radiation compared to the nonradiated baseline (*p* = 0.0125). In addition to the in vivo analysis, we performed ex vivo confocal Raman microscopic imaging of frozen sections derived from the same tumors. A comparison of all control and treated tumors revealed similar trends in the contributions of lipid-like and collagen-like species in both in vivo and ex vivo measurements; however, when evaluated as a function of time, longitudinal trends in the scores of collagen-like and lipid-like components were not consistent between the two data sets, likely due to sample numbers and differences in sampling depth at which information is obtained. Nevertheless, this study demonstrates the potential of fiber-based Raman spectroscopy for identifying early tumor microenvironmental changes in response to clinical doses of radiation therapy.

## Introduction

Radiation therapy is one of the major treatment plans prescribed for patients diagnosed with head and neck squamous cell carcinoma (HNSCC).^1^ The biggest challenge facing these patients is treatment failure due to locoregional recurrence of cancer after therapy. Conventional radiation therapy takes the form of fractionated doses that are spread over several weeks (2 Gy/day; 5 days/week for 6–7 weeks). Treatment response is only evaluated 1–2 months after completion of therapy and is based on Response Evaluation Criteria in Solid Tumors (RECIST), which determines the change in tumor volume post-therapy using clinical imaging modalities, such as X-ray CT and MRI.^2^ There are no methods that can currently identify treatment responders and nonresponders during the treatment regimen. Therefore, there is a significant period of time from the start of therapy to the evaluation of response when patients with nonresponding tumors could be switched to alternative treatment strategies if treatment monitoring approaches were available.

Raman spectroscopy (RS) can provide quantitative and chemically specific information about the biomolecular composition of the tissue. RS is a noninvasive and nondestructive technique that requires minimal sample preparation. RS is based on inelastic scattering of photons after their interaction with biological specimens and allows the quantification of unique vibrational modes of molecules. Thus, biological molecules with unique chemical features can be identified without exogenous dyes, and changes in their content can provide pathological information.^3,4^ Leveraging these advantages, several studies have utilized RS to uncover radiation-induced changes within cells^5,6^ and tissue.^7−9^ Jirasek and colleagues have used RS in a wide range of studies to identify the biomolecular changes within lung and breast tumor xenografts following irradiation and excision.^10−12^ They have shown, both in cell culture and tumor xenograft studies, that RS spectral bands associated with glycogen increase in response to radiation and are linked to radiation resistance.^13−15^ Wu et al. isolated exosomes from radiation-resistant nasopharyngeal carcinoma cells and identified a decrease in spectral features associated with collagen and nucleic acids in the radioresistant exosomes.^16^ Our lab has investigated the ability of Raman spectroscopy to distinguish between radiation-resistant and -sensitive tumors. Using a matched model of radiation resistance and cell lines of known radiation sensitivity, we found statistically significant increases in the contributions of lipid-like and collagen-like species of radiation-sensitive tumors following radiation but no changes in the radiation-resistant tumors following treatment.^17^ However, these studies were performed in tumor xenografts that were excised from animals about 35–50 days following therapy and therefore do not reflect short-term radiation-induced changes within the tumor microenvironment.

**Scheme 1:**
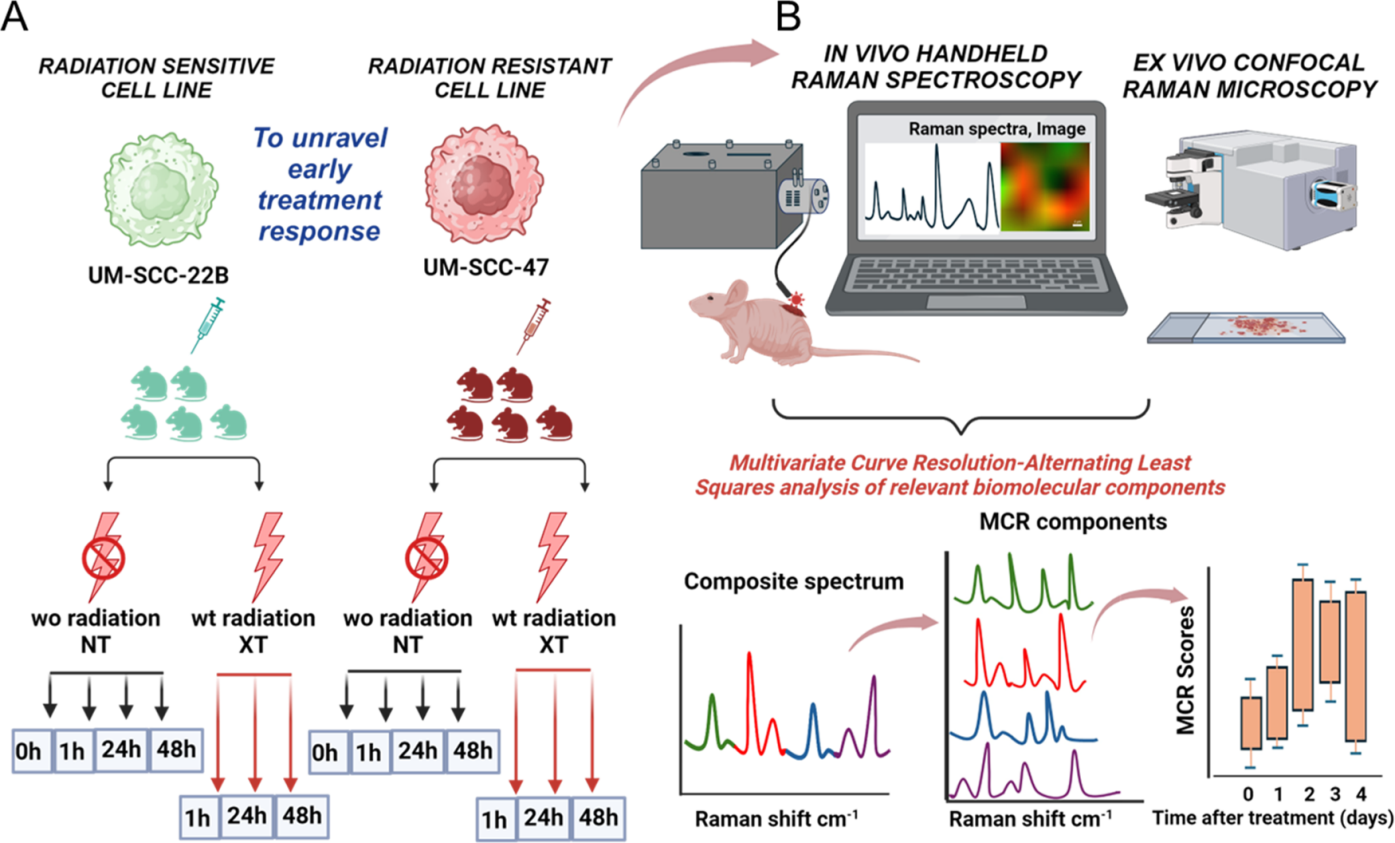
Overall Scheme of the Study (A) Longitudinal monitoring of early treatment response to radiation therapy in tumors derived from two human cancer cell lines UM-SCC-22B (radiation sensitive) and UM-SCC-47 (radiation resistant). The animal groups are classified as without radiation (wo radiation or NT) and with radiation (wt radiation or XT) monitored from 0 hr to 48 hr after radiation therapy. (B) In vivo handheld Raman spectroscopy analysis and ex vivo confocal Raman microscopy analysis of the mice tumor and the sectioned frozen tissue respectively. The composite Raman spectrum was decomposed to obtain pure biomolecule-related MCR components and their corresponding abundance scores. Created with biorender.com.

In this study, we sought to investigate whether short-term biomolecular changes can be observed in radiation-resistant and -sensitive tumors in vivo immediately following irradiation. The overall study schematic is presented in Scheme 1. To this end, we used human head and neck cell lines UM-SCC-22B and UM-SCC-47 with known radiosensitivity to form tumor xenografts. Once tumors reached a volume of 200 mm^3^, they were treated with a single dose of 2 Gy. We performed in vivo handheld Raman spectroscopy on tumors prior to 24 and 48 h following radiation therapy. Using chemometric analysis, we found spectral changes associated with lipid- and collagen-like species in irradiated tumors. Confocal Raman microscopy of tissue sections from the same tumors confirmed our in vivo findings when evaluating all control and treated tumors; however, there were differences in the longitudinal trends of lipid- and collagen-like species between ex vivo and in vivo measurements. These results underline the sensitivity of RS to early radiation-induced biochemical changes in tumor microenvironment and suggest that continuous monitoring of cancer patients undergoing therapy could aid in the identification of nonresponding patients and an improvement in treatment response rates.

## Materials and Methods

### Cell Culture, Tumor Xenografts, and Radiation Treatment

Cell culture conditions have been reported in detail previously.^18,19^ Briefly, UM-SCC-22B and UM-SCC-47 cells were cultured in a mixture of Dulbecco’s modified Eagle medium (DMEM), 10% fetal bovine serum, 1% Penicillin–Streptomycin, 1% nonessential amino acids (NEAA), and 1% l-glutamine. Athymic (nu/nu) mice were purchased from Jackson Laboratories and housed at the Central Laboratory Animal Facility (CLAF) of the University of Arkansas under standard 12 h light/dark cycles with ad libitum access to food and clean water. Animals were allowed to acclimate to animal facility conditions for 2–3 weeks upon their arrival. We formed subcutaneous head and neck tumor xenografts by injecting 1.5 million cells suspended in a 1:1 mixture of Matrigel (Corning, New York) and saline into the right flanks of nude mice. Tumor growth of all mice was monitored daily, and a total of 47 mice were randomly distributed to one of the treatments (control or a single dose of 2 Gy of radiation) and time point (Baseline, 1, 24, and 48 h after radiation) groups once tumor volume reached 200 mm^3^ (See tumor distribution in Table 1).^20^ Treatment of 2 Gy was delivered to the tumor using an X-Rad 320 biological cabinet (Precision X-ray, North Branford, CT). The rest of the animal body was covered using lead blocks. During radiation, mice were kept under anesthesia using a mixture of isoflurane (1.5% v/v) and 100% oxygen. This study was approved by the Institutional Animal Care and Use Committee (IACUC) at the University of Arkansas (Protocol number: 18061). Following in vivo RS measurements at each time point, animals were euthanized, and tumors were excised and snap-frozen for ex vivo analysis. We used 47 mice in total for the in vivo measurements and 44 tumors for the ex vivo measurements. The difference in numbers is primarily due to lower tumor availability for ex vivo measurements at the baseline.

**Table 1:** Tumor Distributions in Different Groups^a^

cell line	treatment	baseline	1 h	24 h	48 h
UM-SCC-22B	NT	7(5)	3	3	2
	XT		2	3	2
UM-SCC-47	NT	4(3)	3	3	3
	XT		4	4	4

a NT and XT, respectively, represent control and radiated animals. For baseline, 7 and 4 mice were used for in vivo spectroscopy in the UM-SCC-22B and UM-SCC-47 tumors, respectively. The numbers in parentheses represent the number of samples used for ex vivo analysis for UM-SCC-22B and UM-SCC-47, respectively.

### Raman Spectroscopy

Prior to in vivo Raman spectroscopic measurements, tumor-bearing animals were anesthetized, and the skin covering the tumor was surgically removed. Great care was taken to prevent damaging the tumor microenvironment and cause unintended bleeding. The bare tumor was brought into contact with the fiber optic probe to acquire Raman spectra. The Raman system used in these studies has been described previously.^17^ Briefly, this system included a diode laser emitting at 830 nm (500 mW maximum power, Process Instruments, Salt Lake City, UT) for excitation and an imaging spectrograph (Holospec f/1.8i, Kaiser Optical Systems, Ann Arbor, MI) with a gold-coated 1200 g/mm grating and a thermoelectrically cooled CCD camera (LS 785, Princeton Acton) for spectral acquisition. The spectral resolution of the Raman spectrometer is 2.33 cm^–1^. A 2 mm flexible, custom-made fiber probe (EmVision LLC, Loxahatchee, FL) was used for light delivery through an excitation fiber located at the center of the probe. The excitation fiber was terminated with a short-pass filter, which transmits laser light and attenuates longer wavelengths. This fiber is surrounded by 15 collection fibers annularly located at approximately 800 μm away from the central excitation fiber. The collection fiber is also preceded by a long-pass filter transmitting tissue Raman spectrum and blocking backscattered light. Finally, a sapphire ball lens was placed after the short- and long-pass filters, creating a distance of 1 mm between the optical fibers and the ball lens to ensure collimation of the excitation light (for reducing the incident energy and preventing tissue damage) and maximal coupling of the in-elastically scattered light into the collection fibers.^21^ The estimated sampling volume achieved by this probe is 1 mm^3^. We acquired 10 RS spectra with an integration time of 1 s/spectrum from 5 locations on each tumor for a total of 50 RS spectra from each tumor and a total of 2350 spectra across all tumors.

### Confocal Raman Microscopy

We used a confocal Raman microscope (XplorRA Plus, JY Horiba, NJ) for all experiments. Frozen sections were removed from −80 °C storage and placed at room temperature for 15 min prior to Raman mapping. Raman spectra were acquired by excitation at 532 nm with an incident laser power of 7.5 mW. Twenty-five (25) spectra (5 × 5 grid) were acquired from 5–7 fields of view with an acquisition time of 3 s. Each field of view corresponds to an area of 30 μm × 30 μm. Spectra were acquired in the range of 400–1800 cm^–1^ (fingerprint region) using a 50× objective and a 1200 lines/mm grating. The spectral resolution of the system is 1.93 cm^–1^.

### Data Analysis

Saturated spectra and spectra contaminated with cosmic rays were visually identified and removed from the data set. Prior to any further processing, the wavenumber axis of the acquired Raman spectra was calibrated by using daily measurements from 4-acetamidophenol. Next, tissue autofluorescence was removed by subjecting the recorded Raman spectra to a fifth-order polynomial fit. Next, the normalized spectra were vector-normalized to neutralize potential variations in laser power and subject to median filtering to remove random spikes induced by cosmic rays to avoid overlap of cosmic rays with biological signal. Following this, the spectra were once again vector-normalized to account for potential changes due to median filtering. We decomposed the spectral data and recovered the spectral profiles (loadings) and the abundance (scores) of the biochemical constituents using multivariate curve resolution-alternating least-squares (MCR-ALS) without prior knowledge of the content of the specimen. MCR-ALS is a well-established method to decompose complex spectra into pure components without prior knowledge of the pure components.^22−24^ We have previously used MCR-ALS in several tumor studies and described it in detail.^17,25−27^ Briefly, composite spectra are decomposed with an iterative optimization routine under non-negativity constraints on both pure spectra and concentration matrices. The model is further constrained to ensure equal lengths of spectra to allow a comparison of the corresponding scores across different groups. The non-negativity constraint helps to solve the complex mixture spectra as loadings and scores representing pure spectra of biochemical constituents and their corresponding abundances, respectively. Based on our prior work, principal component analysis (PCA) was performed on the initial data set because providing these as initial estimates leads to better convergence. The number of output components was chosen empirically, and the same number of principal component analysis (PCA) loadings were presented as initial estimates to the MCR-ALS algorithm. All data preprocessing and analysis were conducted using MATLAB (Mathworks, Natick, MA).

### Statistical Analysis

All statistical analyses were performed using JMP (The SAS Institute, Cary, NC). We used a mixed model to determine the statistical differences in the abundance of different biomolecular species. Tukey HSD tests were used to evaluate significant differences among specific groups. Time (baseline, 1, 24, and 48 h) and treatment (sham control or radiation) were considered fixed effects, while the animal field and view were considered random effects and nested within time and treatment.

## Results

**Figure 1:**
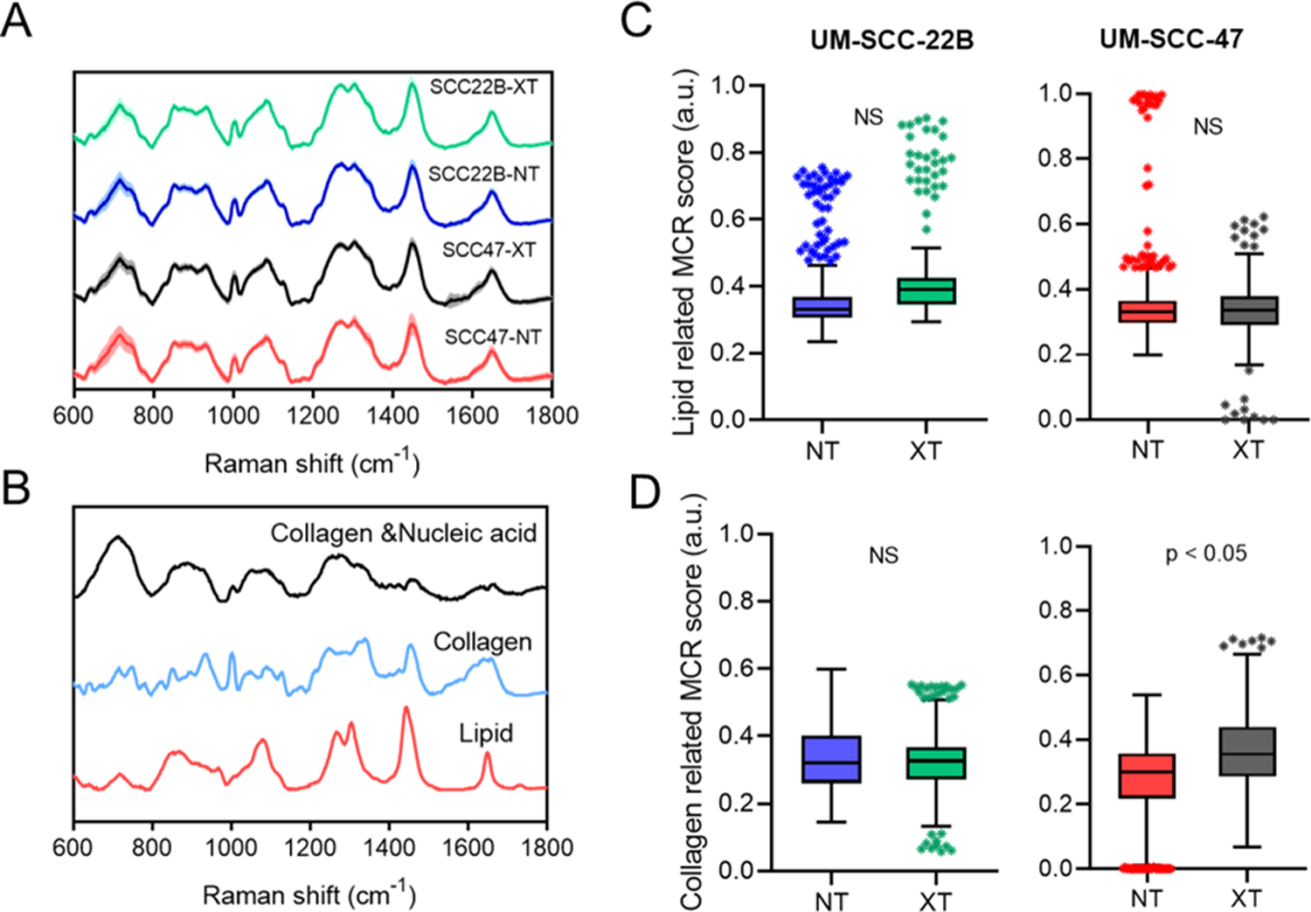
Spectral decomposition of Raman spectra from in vivo samples using the MCR-ALS algorithm. (A) Average Raman spectra ±1 standard deviations from group mean (transparent shadow) collected from NT and XT treated radiation-resistant (UM-SCC-47) and radiation-sensitive (UM-SCC-22B) head and neck tumor xenografts. (B) MCR coefficients derived from raw Raman spectra. Spectra representing lipid-rich, collagen-rich, and a combination of weak collagen and nucleic acid loadings. (C) Boxplots illustrating the scores of lipid-rich, and (D) collagen-rich coefficients in UM-SCC-22B (left panel) and UM-SCC-47 tumors (right panel). Outliers are <10% of data from each group. Significant differences are illustrated by text when *p* < 0.05.

Composite Raman spectra (after preprocessing to remove background autofluorescence and any cosmic ray spikes) acquired from each in vivo tumor group are shown in Figure 1A. Each group consisted of 100–350 spectra, depending upon the number of samples. Regardless of tumor type and treatment, all tumor classes show prominent peaks at ∼715–720 cm^–1^, which are likely due to the C–N stretch of phospholipids (717 cm^–1^) and adenine nucleic acid base (719 cm^–1^), 853 cm^–1^ ring breathing mode of tyrosine and C–C stretch of proline ring, 884 cm^–1^ (collagen), 933 cm^–1^ (collagen), 1002 cm^–1^ (C–C aromatic ring stretching of phenyl alanine), 1085 cm^–1^ (phosphodiester groups in nucleic acids), 1267 cm^–1^ (C–H stretch of lipids/Amide III in collagen), 1301 cm^–1^ (CH vibration of lipids), 1448 cm^–1^ (CH_2_ bending modes in lipids and collagen), and 1656 cm^–1^ (C=C stretching in lipids). Detailed peak assignments and corresponding vibrational modes of these composite spectra are listed in Table S1 (Supporting Information). Although we observed no visually identifiable spectral variations among the four groups, we hypothesized that a subset of wavenumbers representing specific molecular moieties had predictive power but was lost due to averaging. Therefore, we decomposed the Raman spectra using MCR-ALS and obtained three “pure” loadings. Figure 1B illustrates the three MCR loadings that represent key constituents of control (NT) and radiated (XT) UM-SCC-22B and UM-SCC-47 tumors. The lipid-like spectrum (red) contains prominent peaks at 719, 875, 968, 1078, 1268, 1301, 1442, 1656, and 1736 cm^–1^, all of which are characteristic of lipids. The collagen-like spectrum (blue) contains spectral peaks at 752, 823, 853, 937, 1002, 1053, 1128, 1250, 1339, 1454, 1642, and 1661 cm^–1^ which closely resemble characteristics of collagen. The third MCR component (black) contains spectral peaks belonging to a combination of collagen and nucleic acid, which includes 720, 920, 1008, 1060, 1093, 1267, 1335, 1460, 1643, and 1671 cm^–1^. Detailed peak assignments and corresponding vibrational modes of pure spectra are shown in Table S2 (Supporting Information).

In addition to recovering MCR loadings of contributing biological moieties, we extracted the MCR scores that contained the weight (abundance) of each loading for all of the acquired Raman spectra. Using these scores, we quantitatively compared the lipid-like and collagen-like MCR scores of NT and XT tumors from the UM-SCC-22B and UM-SCC-47 groups (irrespective of time points). Even though we observed an increase in the lipid-related MCR scores in the radiated group (XT) of radiation-sensitive UM-SCC-22B tumors, the differences were not statistically significant. Similarly, we found no differences in the MCR scores of lipid-like species (Figure 1C). However, we found a significantly higher contribution from collagen-like species in the treated UM-SCC-47 group compared with that of its sham control (Figure 1D).

**Figure 2:**
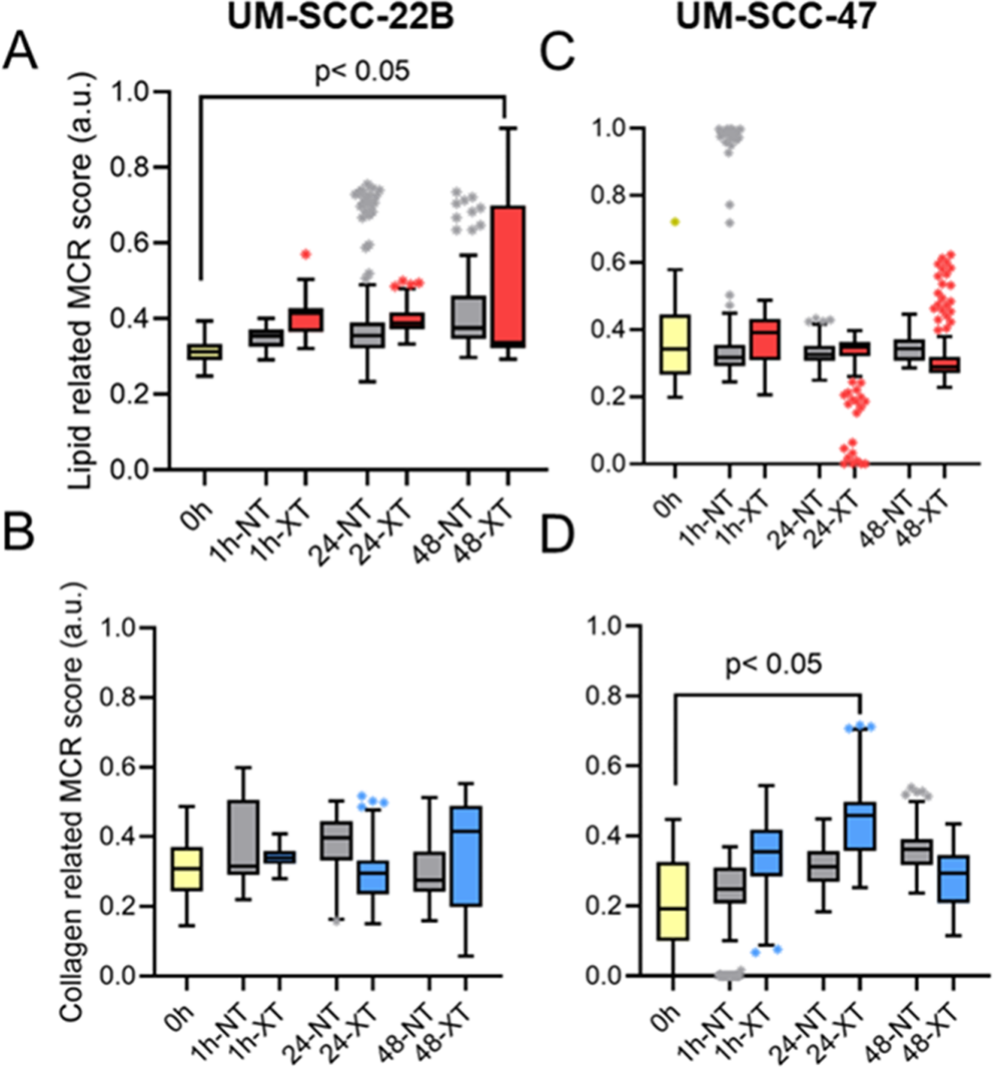
MCR scores of in vivo UM-SCC-22B and UM-SCC-47 tumors at different time points after radiation. Boxplots illustrating the scores of (A), lipid-rich, and (B), collagen-rich coefficients in UM-SCC-22B (left panel) and (C), lipid-rich, and (D), collagen-rich coefficients in UM-SCC-47 tumors (right panel). Outliers in each group are illustrated using asterisks, with the highest percentage of outliers (15%) observed in the 1 h-NT group.

We next sought to identify the biomolecular changes over a period of 48 h following radiation (Figure 2). We observed a statistically significant increase in the MCR score of lipid-like features (*p* < 0.05) in the radiated UM-SCC-22B tumors at 48 h following radiation compared with the baseline group. We also observed a significant increase in the MCR scores of collagen-like features (*p* < 0.05) at 24 h following radiation compared with the baseline group. XT groups at 48 h post radiation had significantly higher values of lipid-related MCR scores with respect to the baseline (*p* < 0.05). In contrast, radiation-resistant UM-SCC-47 tumors did not have significant temporal changes in lipid-related MCR scores between NT and XT groups after radiation time points.

**Figure 3:**
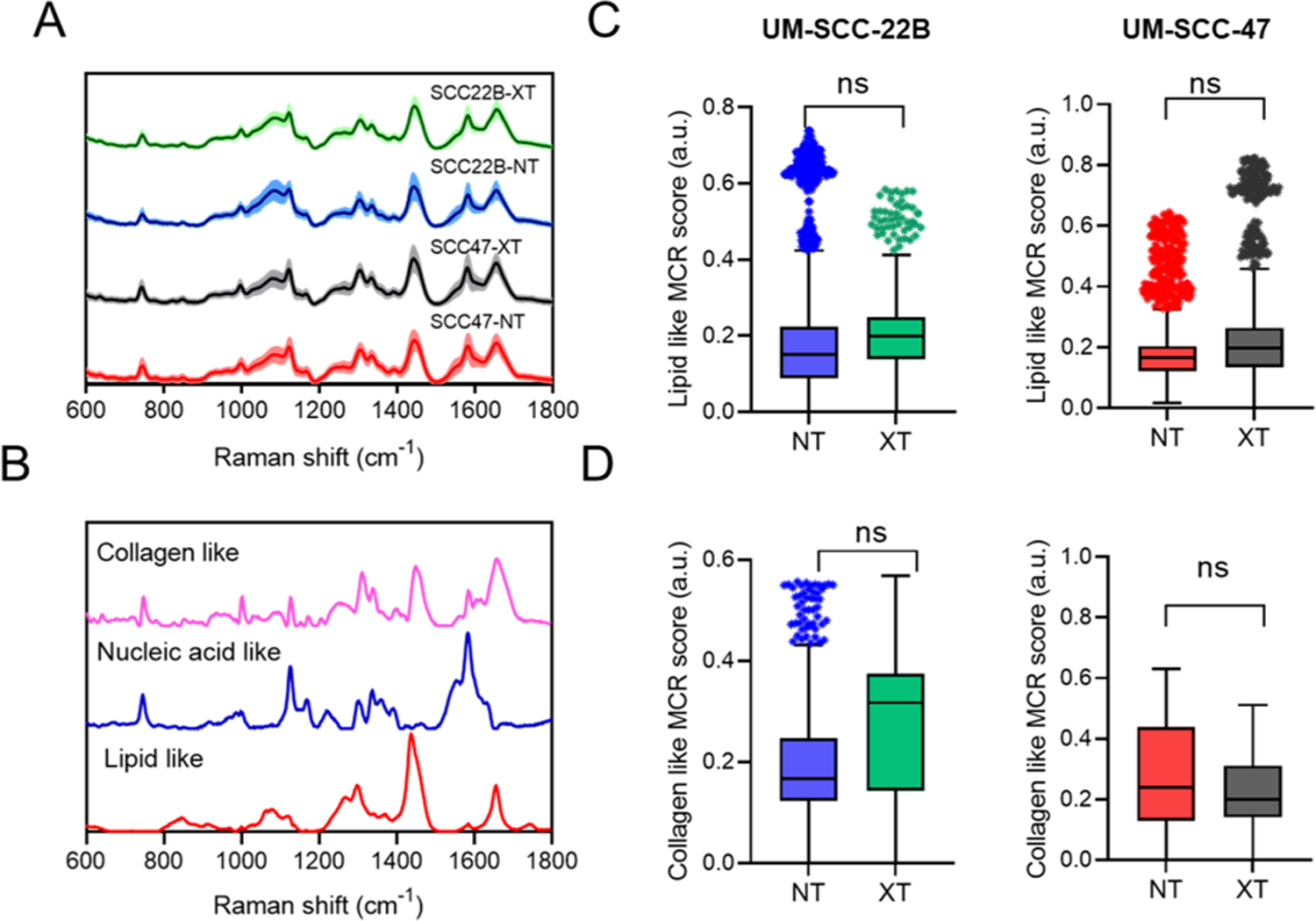
Spectral decomposition of Raman spectra from ex vivo samples using MCR-ALS algorithm (A), average Raman spectra ±1 standard deviations from group mean (transparent shadow) collected from NT and XT treated radiation-resistant (UM-SCC-47) and radiation-sensitive (UM-SCC-22B) head and neck tumors. (B), MCR coefficients derived from raw composite Raman spectra. Red component spectra represent lipid-like loadings, and magenta represents collagen-like loadings. Blue represents a loading that contains spectral features from a nucleic acid. (C), Boxplots illustrating the scores of lipid-rich coefficients in UM-SCC-22B (left panel) and UM-SCC-47 tumors (right panel). (D), Boxplots illustrating the scores of collagen-rich coefficients in UM-SCC-22B (left panel) and UM-SCC-47 tumors (right panel).

Having determined the short-term biomolecular changes within radiated tumors using handheld RS, we used confocal Raman microscopy to obtain RS spectra at high resolution from tumor sections. The goal of these measurements was to determine if the trends observed with volume-averaged optical spectroscopy acquired from multiple locations on the tumor were similar to the RS spectra acquired from multiple fields of view within tumor sections. The preprocessed normalized composite confocal Raman microscopy spectra of different tumor types and treatments are shown in Figure 3A. Each spectral group consisted of approximately 300–750 spectra, depending upon the number of samples in each group. Regardless of tumor type and treatment, all tumor classes show prominent peaks at 746 cm^–1^ (ring breathing mode of DNA/RNA bases), 1002 cm^–1^ (C–C aromatic ring stretching of phenyl alanine), 1070 cm^–1^ (triglycerides (fatty acids), symmetric PO^2–^ stretching of DNA), 1121 cm^–1^ (C–O band of ribose), 1169 cm^–1^ (Tyrosine of Collagen type I), 1304 cm^–1^ (CH_3_, CH_2_ twisting (collagen assignment), CH_2_ deformation (lipid), adenine, cytosine), 1333 cm^–1^ (guanine base in DNA), 1440 cm^–1^ (CH_2_ scissoring vibration), 1579 cm^–1^ (pyrimidine rings of nucleic acids), and 1655 cm^–1^ (C=O stretching lipids and collagen), which forms the composite spectra. Detailed peak assignments and corresponding vibrational modes of composite spectra are depicted in Table S3 (Supporting Information).

Just as in the case of in vivo spectroscopy, we observed no visually identifiable spectral variations among the four groups of frozen sectioned tissue spectra. We decomposed the tissue Raman spectra using MCR-ALS and obtained three “pure” loadings identified as similar to lipid, nucleic acid, and collagen. During MCR analysis, the algorithm occasionally presents duplicate loadings that resemble the existing spectra. In this case, two collagen-like species were identified; the spectrum closest to the established collagen spectrum was used as a pure component. In contrast to in vivo results, here we obtained a pure nucleic acid component in addition to the pure lipid and collagen components. Figure 3B illustrates the three MCR loadings that represent key tumor constituents of the control (NT) and radiated (XT) UM-SCC-22B and UM-SCC-47 tumors. The MCR coefficients corresponding to lipid-like species contain prominent peaks at 1070, 1124, 1270, 1298, 1369, 1440, 1652, and 1738 cm^–1^. The second set of MCR coefficients (blue) contain spectral peaks at 1002, 1128, 1172, 1204, 1252, 1313, 1339, 1401, 1448, 1586, and 1658 cm^–1^, which closely resemble characteristics of collagen. The third MCR component (black) contains spectral peaks belonging to pure nucleic acid, which includes 746, 1120, 1173, 1220, 1304, 1333, 1357, 1424, 1579, and 1630 cm^–1^. Detailed peak assignments and corresponding vibrational modes of each MCR component are shown in Table S4 (Supporting Information).

**Figure 4:**
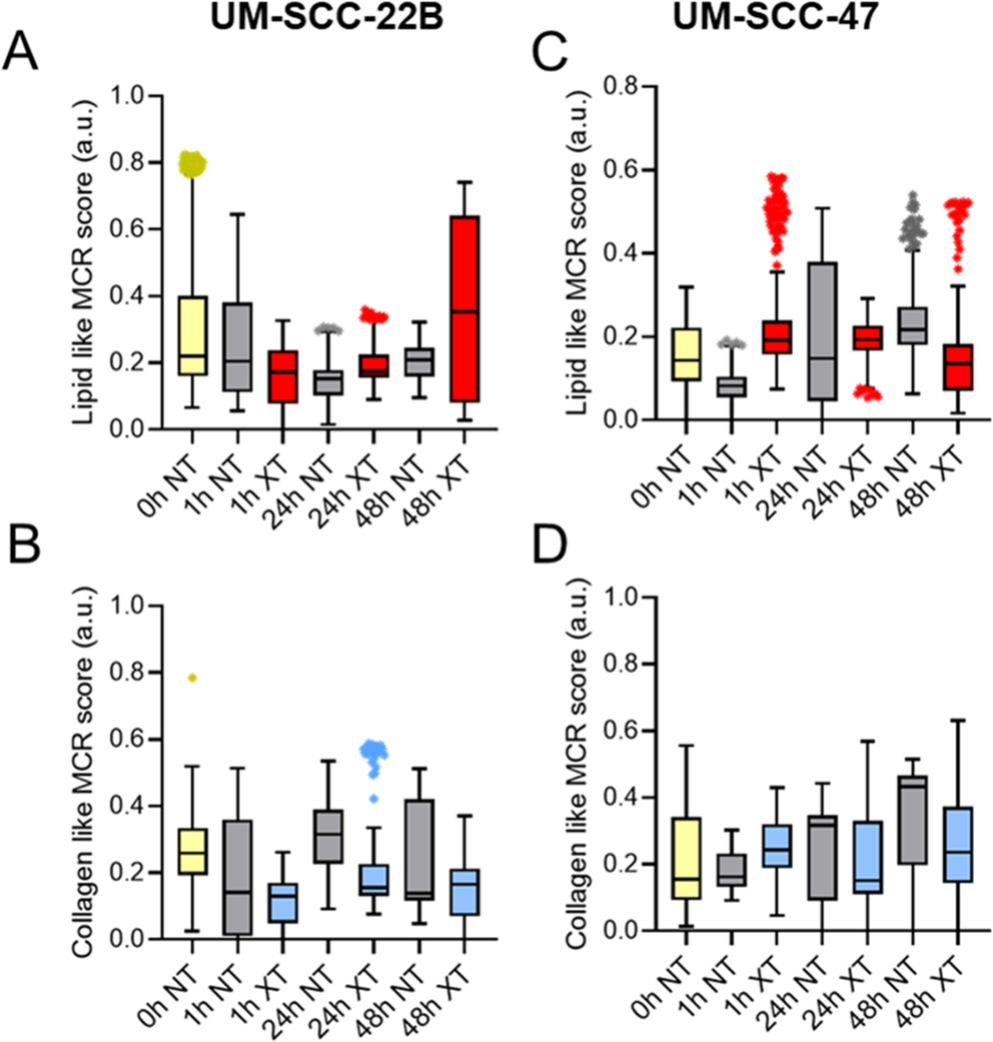
MCR scores of ex vivo UM-SCC-22B and UM-SCC-47 tumors at different time points after radiation. Boxplots illustrating the scores of lipid-rich and collagen-rich species in UM-SCC-22B (A, B) and UM-SCC-47 tumors (C, D).

We evaluated the MCR scores corresponding to lipid-like and collagen-like species to enable a direct comparison to the trends observed with in vivo spectroscopy. There were no significant differences between the NT and XT groups for both cell lines (Figure 3C,D), while the direction of change (from NT to XT) was similar to that of the in vivo results. We also evaluated the longitudinal changes in MCR scores of lipid-like (Figure 4A,B) and collagen-like species (Figure 4C,D) and observed that there were greater inconsistencies in these trends compared to those observed in Figures 1 and 3, likely due to smaller sample numbers. Additionally, unlike the in vivo data set, the changes in lipid-like species in UM-SCC-22B and collagen-like species in UM-SCC-47 were not significantly different. Longitudinal changes in the contributions of nucleic acid-like species are presented in Figure S1A (UM-SCC-22B) and Figure S1B (UM-SCC-47) and were also found to be not significant. We observed that the variance within each group was higher in the ex vivo studies, likely due to the heterogeneity within tumor sections and the smaller fields of view imaged within each section.

## Discussion

Optical technologies continue to be explored for noninvasive, quantitative, and real-time monitoring of treatment response in the clinic.^28^ The ability to evaluate changes in the tumor microenvironment in response to therapy that are associated with treatment resistance can enable improved response rates. Oxygen availability is a critical determinant of resistance to radiation and other therapies.^29^ Several studies have shown that diffuse optical spectroscopy can measure tumor vascular oxygenation in animal models^30−33^ and patients^34−36^ and thus differentiate between treatment responders and nonresponders. Other studies have used two-photon microscopy of endogenous fluorescence from NADH and FAD to reveal that radiation-resistant cells switch to alternative metabolic pathways that enable protection from radiation.^20,37,38^ In addition to such real-time measurements of oxygen supply, demand, and utilization, it is critical to monitor the biomolecular changes within the tumor microenvironment, which are typically assessed only through molecular assays after tumor excision and fixation. Raman spectroscopy offers the ability to monitor dynamic biomolecular shifts within the tumor microenvironment. However, most studies to date have been performed only in cell culture or on excised tumors. Bhattacharjee et al. used RS to determine the in vivo response of rat breast tumors to phototherapy by monitoring the biomolecular changes before treatment and after treatment.^39^ In this study, we sought to test the sensitivity of label-free Raman spectroscopy to early radiation-induced biochemical alterations in vivo head and neck tumors of known radiation sensitivity.

The two dominant species identified in most RS studies and indeed in this study as well are lipids and collagen. Results from both in vivo spectroscopy and ex vivo microscopy identified an increase in the contributions of lipid-like species after radiation therapy; this was especially visible in the radiation-sensitive UM-SCC-22B tumors over a period of 48 h. This is consistent with observations by other groups in radiation-sensitive cell lines^40^ as well as our own work examining radiation-sensitive tumors several weeks after radiation therapy.^17^ Interestingly, our results are also aligned with those of previous studies that demonstrated no changes in lipid-like content in radiation-resistant cell lines and tumors, thereby illustrating a potential early biomarker of radiation sensitivity that can be detected with Raman spectroscopy. De novo lipogenesis has been shown to have a radioprotective effect and thus contributes to radiation resistance in neck cancer cells. Thus, we would have expected an increase in lipid contributions in the radiation-resistant tumors if such changes manifest within such a short time frame. Further work is necessary to understand the exact lipid species that appear to increase in all of these studies and the kinetics of such changes.

Collagen is an important component of the tumor microenvironment that exists in a joint response loop with cancer cells and influences prognosis, progression, metastasis, resistance, and recurrence.^41^ Our studies here demonstrate an increase in collagen-like species about 24 h after radiation therapy in radiation-resistant UM-SCC-47 tumors. Radiation injury is known to incite an acute response by overexpressing growth factors through macrophages that lead to recruitment and development of fibroblasts and myofibroblasts, which leads to collagen secretion.^42,43^ In addition to collagen deposition induced by radiation, collagen has been shown to protect cancer cells against radiation. In vitro studies of renal cell carcinoma have shown that adherence to collagen I protects cancer cells from radiation-induced apoptosis during both normoxic and hypoxic conditions.^44^ Collagen deposition has also been known to be driven by hypoxia-inducible factors (HIFs). In vitro studies of MDA-MB-231 breast cancer cells have shown hypoxia to stimulate prominent collagen cross-linking through HIFs, knockdowns of which abolished collagen cross-linking.^45^ These studies are consistent with our previous findings where we showed that the radiation-resistant UM-SCC-47 tumors have higher content of HIF-1α.^33^ However, as with the lipid results, these studies need to be accompanied by investigations of the specific collagen-like species that appear to increase here.

This study does have some drawbacks. While these measurements were performed in vivo, they were performed in different sets of animals at each time point to enable tumor excision and studies on tumor sections. Monitoring the same group of animals over several days in response to multiple doses of radiation will provide a more rigorous investigation of the biomolecular changes. In addition, future studies should match the depth of sampling of the RS probe used and the depth at which tumor sections are extracted for analysis to enable true one-on-one comparison of spectroscopy and microscopy information. This will also ensure that the trends observed with both techniques are consistent with each other.

## Conclusions

In summary, we have used Raman spectroscopy to observe radiation-induced biochemical alterations in the first 48 h after a single dose of 2 Gy and identified. Multivariate analysis of acquired Raman spectra revealed an increase in lipid-like and collagen-like species in radiation-sensitive and -resistant tumors, respectively. Our future studies include genetic alteration of the pathways that contribute to fatty acid synthesis and collagen breakdown to determine if modification of these pathways leads to corresponding changes in MCR-derived spectral components. Such studies can provide a controlled method to validate the sensitivity of Raman spectroscopy to specific biochemical changes within tissue.

## Supporting Information Available

Detailed peak assignments for each biomolecular component, and MCR scores for nucleic acid contributions within each tumor group (PDF)

## References

[ref1] Rocha P. H. P.; Reali R. M.; Decnop M.; Souza S. A.; Teixeira L. A. B.; Lucas A. Júnior; Sarpi M. O.; Cintra M. B.; Pinho M. C.; Garcia M. R. T. Adverse Radiation Therapy Effects in the Treatment of Head and Neck Tumors. RadioGraphics 2022, 42 (3), 806–821. 10.1148/rg.210150. 35302867

[ref2] Eisenhauer E. A.; Therasse P.; Bogaerts J.; Schwartz L. H.; Sargent D.; Ford R.; Dancey J.; Arbuck S.; Gwyther S.; Mooney M.; Rubinstein L.; Shankar L.; Dodd L.; Kaplan R.; Lacombe D.; Verweij J. New Response Evaluation Criteria in Solid Tumours: Revised RECIST Guideline (Version 1.1). Eur. J. Cancer 2009, 45 (2), 228–247. 10.1016/j.ejca.2008.10.026. 19097774

[ref3] Matthäus C.; Krafft C.; Dietzek B.; Brehm B. R.; Lorkowski S.; Popp J. Noninvasive Imaging of Intracellular Lipid Metabolism in Macrophages by Raman Microscopy in Combination with Stable Isotopic Labeling. Anal. Chem. 2012, 84 (20), 8549–8556. 10.1021/ac3012347. 22954250

[ref4] Tu Q.; Chang C. Diagnostic Applications of Raman Spectroscopy. Nanomedicine 2012, 8 (5), 545–558. 10.1016/j.nano.2011.09.013. 22024196

[ref5] Delfino I.; Perna G.; Lasalvia M.; Capozzi V.; Manti L.; Camerlingo C.; Lepore M. Visible Micro-Raman Spectroscopy of Single Human Mammary Epithelial Cells Exposed to x-Ray Radiation. J. Biomed. Opt. 2015, 20 (3), 035003 10.1117/1.JBO.20.3.035003. 25769498

[ref6] Maguire A.; Vegacarrascal I.; White L.; McClean B.; Howe O.; Lyng F. M.; Meade A. D. Analyses of Ionizing Radiation Effects in Vitro in Peripheral Blood Lymphocytes with Raman Spectroscopy. Radiat. Res. 2015, 183 (4), 407–416. 10.1667/RR13891.1. 25844945

[ref7] Synytsya A.; Alexa P.; Besserer J.; De Boer J.; Froschauer S.; Gerlach R.; Loewe M.; Moosburger M.; Obstova I.; Quicken P.; Sosna B.; Volka K.; Würkner M. Raman Spectroscopy of Tissue Samples Irradiated by Protons. Int. J. Radiat. Biol. 2004, 80 (8), 581–591. 10.1080/09553000412331283515. 15370970

[ref8] Lakshmi R. J.; Kartha V. B.; Krishna C. M.; Solomon J. G. R.; Ullas G.; Devi P. U. Tissue Raman Spectroscopy for the Study of Radiation Damage: Brain Irradiation of Mice. Radiat. Res. 2002, 157 (2), 175–182. 10.1667/0033-7587(2002)157[0175:trsfts]2.0.co;2. 11835681

[ref9] Vidyasagar M. S.; Maheedhar K.; Vadhiraja B. M.; Fernendes D. J.; Kartha V. B.; Krishna C. M. Prediction of Radiotherapy Response in Cervix Cancer by Raman Spectroscopy: A Pilot Study. Biopolymers 2008, 89 (6), 530–537. 10.1002/bip.20923. 18189303

[ref10] Fuentes A. M.; Narayan A.; Milligan K.; Lum J. J.; Brolo A. G.; Andrews J. L.; Jirasek A. Raman Spectroscopy and Convolutional Neural Networks for Monitoring Biochemical Radiation Response in Breast Tumour Xenografts. Sci. Rep. 2023, 13 (1), 1530 10.1038/s41598-023-28479-2. 36707535 PMC9883395

[ref11] Van Nest S. J.; Nicholson L. M.; Pavey N.; Hindi M. N.; Brolo A. G.; Jirasek A.; Lum J. J. Raman Spectroscopy Detects Metabolic Signatures of Radiation Response and Hypoxic Fluctuations in Non-Small Cell Lung Cancer. BMC Cancer 2019, 19 (1), 474 10.1186/s12885-019-5686-1. 31109312 PMC6528330

[ref12] Harder S. J.; Isabelle M.; Devorkin L.; Smazynski J.; Beckham W.; Brolo A. G.; Lum J. J.; Jirasek A. Raman Spectroscopy Identifies Radiation Response in Human Non-Small Cell Lung Cancer Xenografts. Sci. Rep. 2016, 6 (6), 21006 10.1038/srep21006. 26883914 PMC4756358

[ref13] Harder S. J.; Matthews Q.; Isabelle M.; Brolo A. G.; Lum J. J.; Jirasek A. A Raman Spectroscopic Study of Cell Response to Clinical Doses of Ionizing Radiation. Appl. Spectrosc. 2015, 69 (2), 193–204. 10.1366/14-07561. 25588147

[ref14] Matthews Q.; Isabelle M.; Harder S. J.; Smazynski J.; Beckham W.; Brolo A. G.; Jirasek A.; Lum J. J. Radiation-Induced Glycogen Accumulation Detected by Single Cell Raman Spectroscopy Is Associated with Radioresistance That Can Be Reversed by Metformin. PLoS One 2015, 10 (8), e0135356 10.1371/journal.pone.0135356. 26280348 PMC4539228

[ref15] Matthews Q.; Jirasek A.; Lum J. J.; Brolo A. G. Biochemical Signatures of in Vitro Radiation Response in Human Lung, Breast and Prostate Tumour Cells Observed with Raman Spectroscopy. Phys. Med. Biol. 2011, 56 (21), 6839–6855. 10.1088/0031-9155/56/21/006. 21971286

[ref16] Wu Q.; Ding Q.; Lin W.; Weng Y.; Feng S.; Chen R.; Chen C.; Qiu S.; Lin D. Profiling of Tumor Cell-Delivered Exosome by Surface Enhanced Raman Spectroscopy-Based Biosensor for Evaluation of Nasopharyngeal Cancer Radioresistance. Adv. Healthcare Mater. 2023, 12, 2202482 10.1002/adhm.202202482. 36528342

[ref17] Paidi S. K.; Diaz P. M.; Dadgar S.; Jenkins S. V.; Quick C. M.; Griffin R. J.; Dings R. P. M.; Rajaram N.; Barman I. Label-Free Raman Spectroscopy Reveals Signatures of Radiation Resistance in the Tumor Microenvironment. Cancer Res. 2019, 79 (8), 2054–2064. 10.1158/0008-5472.CAN-18-2732. 30819665 PMC6467810

[ref18] Dadgar S.; Troncoso J. R.; Rajaram N. Optical Spectroscopic Sensing of Tumor Hypoxia. J. Biomed. Opt. 2018, 23, 067001 10.1117/1.JBO.23.6.067001. 29873205

[ref19] Stein A. P.; Swick A. D.; Smith M. A.; Blitzer G. C.; Yang R. Z.; Saha S.; Harari P. M.; Lambert P. F.; Liu C. Z.; Kimple R. J. Xenograft Assessment of Predictive Biomarkers for Standard Head and Neck Cancer Therapies. Cancer Med. 2015, 4 (5), 699–712. 10.1002/cam4.387. 25619980 PMC4430263

[ref20] Ivers J. D.; Puvvada N.; Quick C. M.; Rajaram N. Investigating the Relationship between Hypoxia, Hypoxia-Inducible Factor 1, and the Optical Redox Ratio in Response to Radiation Therapy. Biophotonics Discovery 2024, 1 (1), 015003 10.1117/1.BIOS.1.1.015003. PMC11922545 40109884

[ref21] Motz J. T.; Hunter M.; Galindo L. H.; Gardecki J. A.; Kramer J. R.; Dasari R. R.; Feld M. S. Optical Fiber Probe for Biomedical Raman Spectroscopy. Appl. Opt. 2004, 43 (3), 542–554. 10.1364/AO.43.000542. 14765912

[ref22] De Juan A.; Jaumot J.; Tauler R. Multivariate Curve Resolution (MCR). Solving the Mixture Analysis Problem. Anal. Methods 2014, 6 (14), 4964–4976. 10.1039/C4AY00571F.

[ref23] Ando M.; Hamaguchi H. Molecular Component Distribution Imaging of Living Cells by Multivariate Curve Resolution Analysis of Space-Resolved Raman Spectra. J. Biomed. Opt. 2014, 19 (1), 011016 10.1117/1.JBO.19.1.011016. 24108582

[ref24] Felten J.; Hall H.; Jaumot J.; Tauler R.; De Juan A.; Gorzsás A. Vibrational Spectroscopic Image Analysis of Biological Material Using Multivariate Curve Resolution-Alternating Least Squares (MCR-ALS). Nat. Protoc. 2015, 10 (2), 217–240. 10.1038/nprot.2015.008. 25569330

[ref25] Paidi S. K.; Troncoso J. R.; Raj P.; Diaz P. M.; Ivers J. D.; Lee D. E.; Avaritt N. L.; Gies A. J.; Quick C. M.; Byrum S. D.; Tackett A. J.; Rajaram N.; Barman I. Raman Spectroscopy and Machine Learning Reveals Early Tumor Microenvironmental Changes Induced by Immunotherapy. Cancer Res. 2021, 81 (22), 5745–5755. 10.1158/0008-5472.CAN-21-1438. 34645610 PMC8841097

[ref26] Paidi S. K.; Troncoso J. R.; Harper M. G.; Liu Z.; Nguyen K. G.; Ravindranathan S.; Rebello L.; Lee D. E.; Ivers J. D.; Zaharoff D. A.; Rajaram N.; Barman I. Raman Spectroscopy Reveals Phenotype Switches in Breast Cancer Metastasis. Theranostics 2022, 12 (13), 5351–5363. 10.7150/thno.74002. 35910801 PMC9330538

[ref27] Paidi S. K.; Shah V.; Raj P.; Glunde K.; Pandey R.; Barman I. Coarse Raman and Optical Diffraction Tomographic Imaging Enable Label-Free Phenotyping of Isogenic Breast Cancer Cells of Varying Metastatic Potential. Biosens. Bioelectron. 2021, 175, 112863 10.1016/j.bios.2020.112863. 33272866 PMC7847362

[ref28] Dadgar S.; Rajaram N. Optical Imaging Approaches to Investigating Radiation Resistance. Front. Oncol. 2019, 9, 1152 10.3389/fonc.2019.01152. 31750246 PMC6848224

[ref29] Bertout J. A.; Patel S. A.; S M. C. The Impact of O2 Availability on Human Cancer. Nat. Rev. Cancer 2008, 8, 967–975. 10.1038/nrc2540. 18987634 PMC3140692

[ref30] Vishwanath K.; Klein D.; Chang K.; Schroeder T.; Dewhirst M. W.; Ramanujam N. Quantitative Optical Spectroscopy Can Identify Long-Term Local Tumor Control in Irradiated Murine Head and Neck Xenografts. J. Biomed. Opt. 2009, 14 (5), 054051 10.1117/1.3251013. 19895152 PMC2776819

[ref31] Hu F.; Vishwanath K.; Salama J. K.; Erkanli A.; Peterson B.; Oleson J. R.; Lee W. T.; Brizel D. M.; Ramanujam N.; Dewhirst M. W. Oxygen and Perfusion Kinetics in Response to Fractionated Radiation Therapy in FaDu Head and Neck Cancer Xenografts Are Related to Treatment Outcome. Int. J. Radiat. Oncol., Biol., Phys. 2016, 96 (2), 462–469. 10.1016/j.ijrobp.2016.06.007. 27598811 PMC5130289

[ref32] Diaz P. M.; Jenkins S. V.; Alhallak K.; Semeniak D.; Griffin R. J.; Dings R. P. M.; Rajaram N. Quantitative Diffuse Reflectance Spectroscopy of Short-Term Changes in Tumor Oxygenation after Radiation in a Matched Model of Radiation Resistance. Biomed. Opt. Express 2018, 9 (8), 3794–3804. 10.1364/BOE.9.003794. 30338156 PMC6191608

[ref33] Dadgar S.; Troncoso J. R.; Siegel E. R.; Curry N. M.; Griffin R. J.; Dings R. P. M.; Rajaram N. Spectroscopic Investigation of Radiation-Induced Reoxygenation in Radiation-Resistant Tumors. Neoplasia 2021, 23 (1), 49–57. 10.1016/j.neo.2020.11.006. 33220616 PMC7683290

[ref34] Sunar U.; Quon H.; Durduran T.; Zhang J.; Du J.; Zhou C.; Yu G.; Choe R.; Kilger A.; Lustig R.; Loevner L.; Nioka S.; Chance B.; Yodh A. G. Noninvasive Diffuse Optical Measurement of Blood Flow and Blood Oxygenation for Monitoring Radiation Therapy in Patients with Head and Neck Tumors: A Pilot Study. J. Biomed. Opt. 2006, 11 (6), 064021 10.1117/1.2397548. 17212544

[ref35] Dong L.; Kudrimoti M.; Cheng R.; Shang Y.; Johnson E. L.; Stevens S. D.; Shelton B. J.; Yu G. Noninvasive Diffuse Optical Monitoring of Head and Neck Tumor Blood Flow and Oxygenation during Radiation Delivery. Biomed. Opt. Express 2012, 3 (2), 259–272. 10.1364/BOE.3.000259. 22312579 PMC3269843

[ref36] Dong L.; Kudrimoti M.; Irwin D.; Chen L.; Shang Y.; Li X.; Stevens S. D.; Shelton B. J.; Yu G. In Diffuse Optical Measurements of Head and Neck Tumor Hemodynamics for Early Prediction of Radiation Therapy (Conference Presentation), Advanced Biomedical and Clinical Diagnostic and Surgical Guidance Systems XIV; SPIE, 2016; p 8. 10.1117/1.JBO.21.8.085004 PMC4999482 27564315

[ref37] Alhallak K.; Jenkins S. V.; Lee D. E.; Greene N. P.; Quinn K. P.; Griffin R. J.; Dings R. P. M.; Rajaram N. Optical Imaging of Radiation-Induced Metabolic Changes in Radiation- Sensitive and Resistant Cancer Cells. J. Biomed. Opt. 2017, 22 (6), 060502 10.1117/1.JBO.22.6.060502. 28622395 PMC5499259

[ref38] Lee D. E.; Alhallak K.; Jenkins S. V.; Vargas I.; Greene N. P.; Quinn K. P.; Griffin R. J.; Dings R. P. M.; Rajaram N. A Radiosensitizing Inhibitor of HIF-1 Alters the Optical Redox State of Human Lung Cancer Cells in Vitro. Sci. Rep. 2018, 8 (1), 8815 10.1038/s41598-018-27262-y. 29891977 PMC5995847

[ref39] Bhattacharjee T.; Fontana L. C.; Raniero L.; Ferreira-Strixino J. In Vivo Raman Spectroscopy of Breast Tumors Prephotodynamic and Postphotodynamic Therapy. J. Raman Spectrosc. 2018, 49 (5), 786–791. 10.1002/jrs.5360.

[ref40] Deng X.; Ali-Adeeb R.; Andrews J. L.; Shreeves P.; Lum J. J.; Brolo A.; Jirasek A. Monitor Ionizing Radiation-Induced Cellular Responses with Raman Spectroscopy, Non-Negative Matrix Factorization, and Non-Negative Least Squares. Appl. Spectrosc. 2020, 74 (6), 701–711. 10.1177/0003702820906221. 32098482

[ref41] Xu S.; Xu H.; Wang W.; Li S.; Li H.; Li T.; Zhang W.; Yu X.; Liu L. The Role of Collagen in Cancer: From Bench to Bedside. J. Transl. Med. 2019, 17 (1), 309 10.1186/s12967-019-2058-1. 31521169 PMC6744664

[ref42] Li M.; Jendrossek V.; Belka C. The Role of PDGF in Radiation Oncology. Radiat. Oncol. 2007, 2 (1), 5 10.1186/1748-717X-2-5. 17217530 PMC1780053

[ref43] Yarnold J.; Vozenin Brotons M. C. Pathogenetic Mechanisms in Radiation Fibrosis. Radiother. Oncol. 2010, 97 (1), 149–161. 10.1016/j.radonc.2010.09.002. 20888056

[ref44] Krasny L.; Shimony N.; Tzukert K.; Gorodetsky R.; Lecht S.; Nettelbeck D. M.; Haviv Y. S. An In-Vitro Tumour Microenvironment Model Using Adhesion to Type i Collagen Reveals Akt-Dependent Radiation Resistance in Renal Cancer Cells. Nephrol., Dial., Transplant. 2010, 25 (2), 373–380. 10.1093/ndt/gfp525. 19828461

[ref45] Wong C. C. L.; Gilkes D. M.; Zhang H.; Chen J.; Wei H.; Chaturvedi P.; Fraley S. I.; Wong C. M.; Khoo U. S.; Ng I. O. L.; Wirtz D.; Semenza G. L. Hypoxia-Inducible Factor 1 Is a Master Regulator of Breast Cancer Metastatic Niche Formation. Proc. Natl. Acad. Sci. U.S.A. 2011, 108 (39), 16369–16374. 10.1073/pnas.1113483108. 21911388 PMC3182724

