# Supplementary material for: Investigating In Vivo Tumor Biomolecular Changes Following Radiation Therapy Using Raman Spectroscopy
Source: ACS Omega. 2024 Oct 9;9(42):43025–33. doi: 10.1021/acsomega.4c06096 (PMC11500151; doi:10.1021/acsomega.4c06096)
Supplement: Supplementary file 1 — ao4c06096_si_001.pdf [file ao4c06096_si_001.pdf]

SUPPORTING INFORMATION

Investigating in vivo tumor biomolecular changes following radiation therapy using Raman spectroscopy

Varsha Karunakaran[‡,1] *Sina Dadgar*[‡,1] *Santosh K. Paidi,*[2] *April F. Mordi,*[1] *Whitney A. Lowe,*[1] *Umme Marium Mim,*[1] *Jesse D. Ivers,*[1] *Joel I. Rodriguez Troncoso,*[1] *Jared A. McPeake,*[1] *Alric Fernandes,*[1] *Sanidhya D. Tripathi,*[1] *Ishan Barman,*[2] *and Narasimhan Rajaram*[1,*]

[1]Department of Biomedical Engineering, University of Arkansas, Fayetteville, Arkansas, 72701

[2]Department of Mechanical Engineering, Johns Hopkins University, Baltimore, Maryland, 21218

* Corresponding author

List of contents:

Sl. No	Contents
Table S1	Peak assignments and corresponding vibrational modes of handheld Raman spectroscopy composite spectra
Table S2	Peak assignments and corresponding vibrational modes of handheld Raman spectral data of pure spectra of MCR components
Table S3	Peak assignments and corresponding vibrational modes of confocal Raman microscopy composite spectra
Table S4	Peak assignments and corresponding vibrational modes of confocal Raman spectral data of pure spectra of MCR components
Figure S1	Nucleic acid component score box plot from confocal Raman microscopy
Figure S2	Characterization of gold nanoparticles

Table S1. Peak assignments and corresponding vibrational modes of handheld Raman composite spectra [1]

Wavenumber (cm^{-1})	Tentative metabolite	Raman Vibrational modes
715-720	Lipids/ Nucleic acid	C-N (membrane phospholipids head)/Adenine
853	Amino acids	Ring breathing mode of tyrosine & C-C stretch of proline ring
884	Collagen	Proteins, including collagen I
933	Collagen	Proline, hydroxyproline, v(C-C) skeletal of collagen backbone
1002	Phenyl alanine	C-C aromatic ring stretching
1085	Nucleic acid	Phosphodiester groups
1267	Lipid and Collagen	C-H (lipid in normal tissue), Amide III (collagen assignment)
1301	Lipids	CH vibration
1448	Lipids and Collagen	CH_2 bending modes
1656	Lipids	C = C stretching

Table S2. Peak assignments and corresponding vibrational modes of handheld Raman spectral data of pure spectra of derived MCR components [1]

COLLAGEN	
**Wavenumber (cm^{-1})**	**Raman vibrational modes**
752	Symmetric breathing of tryptophan (protein assignment)
823	Out-of-plane ring breathing, tyrosine (protein assignment)
853	Ring breathing mode of tyrosine & C-C stretch of proline ring
937	C-C vibration of collagen backbone
1002	C-C aromatic ring stretching Phenylalanine
1053	C-O stretching, C-N stretching (protein)
1128	C-N stretching (proteins)
1250	Amide III
1339	CH_2/CH_3 wagging of collagen
1454	CH_3 bending & CH_2 scissoring of collagen
1642	Amide I
1661	Amide I

LIPID	
**Wavenumber (cm^{-1})**	**Raman vibrational Modes**
719	Phospholipids
875	Phospholipids
968	Lipids
1078	C-C or C-O stretching mode of phospholipids
1268	d (=C-H) (phospholipids)
1301	C-H vibration (lipids)
1442	Fatty acids, CH_2 bending mode
1656	C=C (lipids)
1736	C=O ester (lipids)

COLLAGEN & NUCLEIC ACID	
**Wavenumber (cm^{-1})**	**Raman vibrational Modes**
720	DNA
920	C-C stretch of proline ring (collagen)
1008	v(C-C) phenylalanine
1060	PO_2^- stretching (DNA/RNA)
1093	Symmetric PO_2^- stretching vibration of the DNA
1267	Amide III (collagen assignment)
1335	CH_3CH_2 wagging, collagen (protein assignment)<br>CH_3CH_2 wagging, nucleic acid
1460	CH_2/CH_3 deformation of lipids & collagen, CH_2 wagging, CH_2/CH_3 deformation<br>Deoxyribose
1643	Amide I band
1671	Amide I

Table S3. Peak Assignments and Corresponding Vibrational Modes of Confocal Raman Spectral Data of Composite Spectra [1]

Wavenumber (cm^{-1})	Tentative metabolites	Raman vibrational modes
746	DNA/RNA	T (ring breathing mode of DNA/RNA bases)
1002	Phenyl alanine	C-C aromatic ring stretching
1070	Lipid, DNA	Triglycerides (fatty acids), Symmetric PO_2^- stretching of DNA
1121	Ribose	C-O band
1169	Collagen Type I	Tyrosine
1304	Collagen, Lipid, DNA	CH_3, CH_2 twisting (collagen assignment), CH_2 deformation (lipid), adenine, cytosine
1333	DNA	Guanine

1440	Lipid	CH_2 scissoring vibration
1579	DNA	Pyrimidine rings (Nucleic acids)
1655	Lipids and Collagen	C=O stretching lipids and collagen

Table S4. Peak Assignments and Corresponding Vibrational Modes of Confocal Raman Spectral Data of pure Spectra of of derived MCR components [1]

**COLLAGEN**	
**Wavenumber (cm^{-1})**	**Vibrational modes**
1002	Phenylalanine
1128	C-N stretching
1172	d(C-H), tyrosine
1204	Amide III
1252	Amide III
1313	CH_3CH_2 twisting mode of collagen
1339	CH_2/CH_3 wagging of collagen
1401	Bending modes of methyl groups in collagen
1448	Collagen
1586	Phenylalanine, hydroxyproline
1658	Amide I band

**LIPID**	
**Wavenumber (cm^{-1})**	**Vibrational Modes**
1070	Triglycerides (fatty acids)
1124	v(C-C) skeletal of acyl backbone in lipid (transconformation)
1270	Typical Phospoholipids
1298	Fatty acids
1369	Lipids
1440	CH_2 scissoring vibration (lipid band)
1652	Lipid (C=C stretch)
1738	Lipids

**NUCLEIC ACID**	
**Wavenumber (cm^{-1})**	**Vibrational Modes**
746	T (ring breathing mode of DNA/RNA bases)
1120	The strong C-O band of ribose
1173	Cytosine, guanine
1220	T,A (DNA/RNA)
1304	Adenine, cytosine

1333	Guanine
1357	Guanine
1424	Deoxyribose
1579	Pyrimidine ring nucleic acid
1630	In-plane double end vibrations of bases

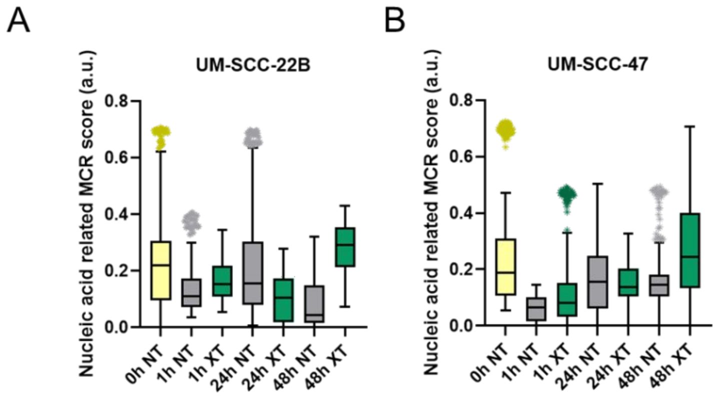

Figure S1: MCR scores of UM-SCC-22B and UM-SCC-47 tumors at different time points after radiation. Boxplots illustrating the scores of nucleic acid-rich coefficients in A. UM-SCC-22B (upper left panel) and B, in UM-SCC-47 tumors (right panel). Differences are found to be non-significant (ns) for nucleic acids.

References

[1] A.C.S. Talari, Z. Movasaghi, S. Rehman, I. ur Rehman, Raman Spectroscopy of Biological Tissues, Appl. Spectrosc. Rev. 50 (2015) 46–111.
